# How to improve the assessment of BPM maturity in the era of digital transformation

**DOI:** 10.1007/s10257-021-00549-w

**Published:** 2021-12-27

**Authors:** Marek Szelągowski, Justyna Berniak-Woźny

**Affiliations:** Systems Research Institute of the Polish Academy of Sciences, Newelska 6, 01-447 Warsaw, Poland

**Keywords:** Business process management (BPM), BPM maturity models (BPM MM), Process nature, Industry 4.0, Digital transformation

## Abstract

For almost 30 years, the way of building business process management maturity models (BPM MMs), the importance assigned to individual maturity levels, and the criteria and critical success factors chosen for BPM maturity assessment have not changed significantly, despite the fact that during those three decades, the business environment and organizations themselves have changed enormously. The impact of hyperautomation and the increasing pace of change require the integration of maturity assessment with the BPM implementation methodology, including the repetition of maturity assessment for selected groups of processes. This causes an urgent need to adapt both process maturity assessment methods and BPM MMs to changing working conditions and business requirements. This conceptual paper is based on a model approach. The framework presented in the article continues and at the same time clearly deviates from the tradition of building BPM MMs on the basis of the Capability Maturity Model (CMM). It proposes a two-stage comprehensive process of organizational process maturity assessment, fully integrated into the process of BPM implementation and further business process management. The presented framework makes it possible to assess the process maturity of Industry 4.0 organizations in which dynamic knowledge-intensive business processes (kiBPs) play a key role in creating value.

## Introduction

Business process management (BPM) has so far been the driving force behind the optimization and the growth of the operational efficiency of companies (Davis and Brabänder 2007; Dumas et al., 2018). Industry 4.0, in which are embedded systems, semantic machine-to-machine communication, IoT and CPS technologies integrating virtual space with the physical world, as well as a new generation of industrial systems such as smart factories (GTAI 2014; Xu et al. 2018), has brought changes in various areas of the organization's functioning through digital transformation, including changes in combination with other transformations (for example green or social). The high speed and parallelism of many change processes generate dynamics and complexity that make it difficult or even impossible to reliably forecast and plan (Leimstoll et al. 2018). Therefore, the current business environment and competitive factors also, and perhaps above all, require that business (especially e-business) and its processes be flexible and responsive. Thus, BPM cannot focus solely on classical planning techniques – it requires a holistic approach that also takes into account the social aspects of the business environment, such as corporate strategies, organizational policies, communication, and cooperation (Kir and Erdogan 2021). However, such an approach requires understanding the nature of business processes (BPs) from the point of view of their unpredictability and knowledge intensity, which in turn allows for their correct classification and provides the basis for their more accurate and effective management.

Business Process Management Maturity Models (BPM MM) are used to determine the current status of the implementation of BPM in an organization and are the basis for planning and increasing its effectiveness (Couckuyt and Van Looy 2020; Van Looy et al. 2017; Tarhan et al. 2015; Van Looy 2013; Roeglinger et al. 2012; Poeppelbuss and Roeglinger 2011; Gottschalk 2009; Becker et al. 2009; de Bruin et al. 2005). Incorrect assessment of maturity means that both the ongoing management based thereon and the implementation of the organization's development do not fully use its potential and may even inhibit this development or, in extreme cases, even pose a threat to the existence of the organization. Therefore, the selection of the appropriate BPM MM or another method of assessing process maturity plays a key role in building competitive advantage. Digital transformation, additionally accelerated by the COVID-19 pandemic, visibly changed the work and social culture, expanded the use of remote working tools, and gave additional impetus to hyperautomation (Rodrigues et al. 2021; Sharma et al. 2020). As a result, an increasing number of often traditional, repetitive business processes are reduced to tasks performed automatically, without human intervention (Gartner 2019). At the same time, dynamic processes that are difficult to copy and which create value through the use of the knowledge and creativity of an organization’s employees, are growing in importance for the organization. Employment in knowledge-intensive activities in the EU already amounts to 36.2% (Eurostat 2021). In countries such as Belgium, Ireland, and Switzerland, this indicator exceeds 44% (Eurostat 2021). Therefore, the development of the organization aimed at adapting it to the changes taking place in the entire business ecosystem makes it necessary to reformulate the requirements in relation to the BPM MM, not only with regard to the key success factors built into it, but also with regard to flexibility, speed allowing for frequent assessment, and ease of application (Bispo et al. 2019; Van Looy 2013; Roeglinger et al. 2012; Rosemann et al. 2004).

Unfortunately, many business organizations do not use or even find themselves falling behind in digital transformation due to the lack of effectively designed business and e-business processes – in the US retail industry alone, almost $40 billion is lost annually because of inadequate or lacking digitized inter-firm business process operations (Zhu et al. 2015). The lack of well-designed processes and a reliable assessment of the BPM maturity of the organization make it impossible to effectively choose and implement integrated business software systems, which are required to be flexible, allow for efficient management, and increase productivity (Leimstoll and Quade 2016). However, development of the BPM MM has not kept up with changes in the entire business ecosystem. Research shows that in the last three decades, the development of process maturity models has been limited to duplicating and detailing changes to the logic of the Capability Maturity Model (CMM) developed by the Software Engineering Institute at Carnegie Mellon University (Paulk et al. 1993). The names, number of the maturity levels, and critical success factors are changing and new variants are developed for various industries or sizes of the organization, but the BPM MM is still based on the traditional understanding of business processes as optimal sequences of activities, prepared in detail by the management of the organization, who know how the processes will have to be executed long before their execution (Dumas et al. 2018; Krogstie 2016; Rosemann et al. 2006). Most of them take into account only or almost exclusively evaluation criteria limited to traditional business processes (Szelągowski and Berniak-Woźny 2019; Andriani et al. 2018; Zare et al. 2018; AlShathry 2016; Chaghooshi et al. 2016; Shafiei and Hajiheydari 2014; Figueiredo et al. 2014; Rohloff 2009). As a result, the assessment of organizational process maturity provided by managers does not take into account most of the Industry 4.0 organizational value-creating processes that require dynamic management (Olding and Rozwel 2015). Depending on the nature of the organization's BPs, this may either turn out to be a minor, negligible inaccuracy (for an organization delivering value through the implementation of predictable and fully repeatable processes) or give a completely false, misleading assessment (for an organization creating value thanks to unique knowledge-intensive BPs (kiBPs)). This causes an urgent need to evaluate the current BPM MMs and adapt them to the requirements of the business environment under the digital transformation. Thus, the research aim of this article is to build a two-stage theoretical framework of the BPM maturity assessment process integrated into BPM implementation, as well as one which meets the requirements of the digital transformation.

The article is structured as follows: Sect. 2 of the article presents the applied methodological approach. Section 3 presents the current view on business process management (BPM), changing critical success factors (CSF), and the business process maturity models (BPM MMs) to formulate the key limitations of BPM MMs from the perspective of the current business environment requirements. In Sect. 4, the authors present results of the search for the concept for BPM MM adaptation that responds to the previously presented limitations. The Conclusions section summarizes the key research results and discusses the prospects of its further development.

## Methodology

The article is conceptual in nature. Traditionally, concept articles can use four approaches – theory synthesis, theory adaptation, typology-based approaches, and model-based approaches (Jaakkola 2020). This conceptual article is based on a model approach that identifies new, previously unexplored relationships between concepts and their individual elements to obtain the expected result (Cornelissen 2017). Authors are not constrained by data limitations, which allows them to explore and model phenomena where little empirical data is available (Yadav 2010).

The aim of the research is to build a theoretical framework for the BPM maturity assessment process integrated with the implementation of BPM and responding to assumptions for the process maturity model in accordance with the requirements of digital transformation. The research approach is based on two stages: (1) a literature study to identify the most important limitations of available BPM MMs in the context of digital transformation requirements that need to be eliminated, and (2) development of a theoretical framework of the BPM maturity assessment process integrated into BPM implementation, as well as one which meets the requirements of digital transformation. Authors use deductive reasoning to explain relationships between key variables based on the concepts discussed previously (MacInnis 2011).

## Literature review

### Diverse nature of the organization's business processes

For over 10 years, both management researchers and practitioners have recognized the diversity of the nature of business processes implemented by organizations operating in Industry 4.0 (Szelągowski 2014; van der Aalst 2013). In accordance with the works of van der Aalst et al. (2005), Van Looy et al. (2011), Kemsley (2011), and Di Ciccio et al. (2012) BPs can be divided according to the dynamics of execution into: (1) structured, (2) structured with ad hoc exceptions, (3) unstructured with pre-defined fragments, and (4) unstructured. As research shows, traditionally-understood structured, repetitive BPs now account for only about 30% of all organizational processes (Olding and Rozwell 2015). This percentage is constantly decreasing due to their ease of automation and their importance for gaining or maintaining the competitive advantage of the organization is constantly decreasing due to the ease of copying them by competitors (Szelągowski and Berniak-Woźny 2019; Olding and Rozwell 2015; Pucher 2012). Unpredictable, knowledge-intensive business processes, which are difficult to copy, are gaining increasing importance (Szelągowski 2021).

The diversification of the nature (the inherent features) of BPs and the growing importance of non-traditional business processes require different methods of implementing and using BPM. Case management methods are not suitable for structured processes while modeling process execution as a sequence of tasks is not a way to improve unstructured processes. Processes of diverse nature also require the use of various hyperautomation technologies to support the execution of processes (Gartner 2019). RPA tools cannot be applied to kiBPs (Gartner 2021; Forrester 2019), while using artificial intelligence (AI) for fully repeatable static processes is non-productive. When assessing BPM maturity in order to prepare a BPM implementation or to improve an implemented BPM, organizations should first assess the nature of their BPs and then assess BPM maturity in a manner consistent with the said nature. Other criteria should be assessed for traditional BPs, for which, at the design or implementation stage, the organization has full knowledge of their course, the process roles that implement them, the necessary documentation, and the expected values of performance measures. In this case, the process maturity of the organization is determined by the quality of the description of business processes, the optimal design of their course, accuracy and repeatability of execution, or the ability to improve processes in accordance with the Deming Cycle (Deming 1986; Szelągowski and Berniak-Woźny 2019), because they determine the value provided by each BP. However, these are not factors that determine efficiency or competitive advantage in the case of unpredictable kiBPs. The value provided by these processes depends not on the perfect repetition of a previously-designed sequence of tasks, but on the use of the knowledge and dynamism of knowledge workers who implement them, within the often unique context of process implementation (Couckuyt and Van Looy 2020; vom Brocke et al. 2014).

In organizations embarking on digital transformation, the most critical business processes are often very complex—spanning many separate departments. As numerous studies show, the use of BPM may become a key driver of the digital transformation of organizations around the world (Butt 2020). For this to happen, however, various processes will require different management principles, resources, and supporting information (IT) systems, as these policies and resources vary widely between structured and unstructured processes. Examples of management principles and resources necessary for various processes are presented in Table 1.

**Table 1:** Sample management principles and resources necessary for processes of various natures

Management principles and resources	Structured BPs	Unstructured BPs
Principles of process execution	Delivering value by accurately repeating the designed process	Providing value by using the dynamism and knowledge of employees, in accordance with the context of execution
Goals and principles of process modeling and design	The optimal required sequence of tasks to be performed, defined with process executors in mind	Defined BP goals, the ontology of available resources (possibilities and limitations) and predefined tasks recommended for execution
Principles of process improvement	Formal, organizational, exceeding the process execution time, usually periodically repeated in accordance with the Deming Cycle	Dynamic shaping of the process flow by its contractors in accordance with the context of implementation, exceeding the process execution time, and improvement based on data on actual performance discovered from actual performances using e.g., process mining
Applied IT tools and solutions	Modelers, workflows, RPA	iBPMS, process mining, ML/ AI
Requirements for people performing the process	Accuracy and efficiency of operation in accordance with the optimal pattern	Innovation and creativity according to context of execution including available supporting technologies

Source: Authors' own elaboration

Therefore, for processes of various natures, different criteria for the assessment of process maturity should be used, because the value provided by them is determined by various critical success factors (CSF).

### The need to adapt the principles of BPM maturity analysis to the nature of the implemented business processes

Critical success factors (CSFs) in BPM are the areas impacting or even determining the success of BPM implementation (Ariyachandra and Frolick 2008; Rosemann and de Bruin 2005b; Bandara et al. 2005). Gates (2010) defines CSFs as “the handful of key areas where an organization must perform well on a consistent basis to achieve its mission.” Similarly, Dabaghkashani et al. (2012) define success factors as those key areas where “things must go right” in order for the BPM initiative to proceed efficiently and be completed successfully. When defining CSFs, one should also take into account the barriers that affect the success of BPM implementation, such as resistance to change, lack of understanding of BPM principles, and lack of consistency of the organization-wide BPM approach (Rosemann et al. 2004). CSFs can be used to better adjust the systematic management of processes in an organization, as they represent the areas that should be the subject of constant and careful management attention (Dobbins and Donnelly 1998). A thorough understanding of CSFs allows the organization to assess its threats, opportunities, weaknesses, and strengths in the process of implementing business process management in the organization and determining the optimal use of this approach to achieve its strategic goals.

Defining practical CSFs for BPM requires a decomposition of a holistic view of specific areas, which would enable a more explicit analysis (Rosemann and vom Brocke 2015). Several papers sought to identify CSFs of BPM (e.g., Reijers and Kohlbacher 2012; Dabaghkashani et al. 2012; Trkman 2010; McCormack et al. 2009; Ariyachandra and Frolick 2008; Hammer 2007; Bandara et al. 2005; Fisher 2004). Unfortunately, there are only a few studies that aim to establish or validate the relationship between critical success factors and approaches to implementing BPM (Bispo et al. 2019). Moreover, few of these studies provide robust methods that can systematically analyze, evaluate, and model an organization's readiness to implement BPM. Additionally, the study Buh et al. 2015 shows that the identification of the indicated CSFs for BPM adoption only provides a limited picture, as the factors change between stages and organizations need to accurately define the stage and prepare a BPM implementation plan.

There are a number of research studies aimed at identifying and categorizing critical success factors that influence BPM implementation in general and at various stages of BPM adoption (Tarhan and Turetken 2016; Buh et al. 2015; Bai and Sarkis 2013; Hajiheydari and Dabaghkashani 2011). Based on this, we can divide BPM CSF into two categories: organization-specific and BPM-specific (Table 2).

**Table 2:** Characteristics of the CSF used in BPM MMs

CSF	Characteristic	KPI Definition Areas
*Organization-specific CSFs*		
Strategic alignment	The goals of BPM implementation are aligned with the strategic goals of the organization. Thanks to this alignment, the results of BPM initiatives are related to business goals and gain importance	Goals of BPM implementations aligned with strategic goals
Governance	Governance is about creating the right structures, indicators, roles, and responsibilities to measure and manage an organization's performance, including the optimization and sustainability of BPM implementation in an organization	Governance of process initiatives; Well-defined accountability; business environment
Management commitment and involvement	High-level management is committed to and involved in BPM initiatives, which translates into creating motivation for implementation, obtaining the required resources and ensuring strategic alignment	Leadership; management accountability
Information and communication technologies (ICT) infrastructure	ICT infrastructure is a combination of common hardware, software and network solutions together with policies and rules for maintaining these solutions, thus supporting the execution and management of business processes	Integration of process and data; automation; BPM suites/systems; deployment; level of ICT investment
Organization culture	This term pertains to the values and behaviors that influence the social and psychological environment specific to an organization. Organizational culture represents the collective values, beliefs, and principles of the members of the organization and therefore plays a significant role in the success of BPM efforts	Collaborative working environment; culture of change; synergies between different departments; bureaucracy of the domain
Stakeholder involvement	Process management is based on a network of relationships between internal and external stakeholders necessary for the adaptation of organizational processes to the dynamic business environment	End user participation; stakeholders’ analysis; stakeholders’ communication
Skilled and empowered employees	This pertains to the skillset required for existing process and those required by individuals to support their performance in the newly defined processes. Empowered employees can take decisions independently, which may result in smoother operations with shorter throughput times	Capable and motivated employees; training and development employee commitment; employee empowerment; employee motivation and rewarding mechanisms
Change management and communication:	Change management refers to significant changes in an organization to improve its performance. Changes can affect processes, technology, personnel, organizational structure and culture and should be transparent and effectively communicated throughout the organization	Readiness for change; re-organization of information; experience with change management; communication between process team and organizational staff
*Business process management specific CSFs*		
Process management organization	Process-based organization of BPM implementation is important for effective planning and maintenance of BPM efforts	Process management structure; establishing a suitable team organization; appointment of process owners; well-organized maintenance and control of the process models
Process management competencies	An organization’s background in BPM knowledge and skills is an important indicator of its competency in BPM implementation	ability to redesign business processes; competencies of BPM team; training and re-skilling; using external consultants
Domain (Process) understanding	Prevalence of domain understanding is crucial in support of process management organization and competencies	Awareness and understanding of the process by employers; level of employee specialization; understanding interdependencies of data sources
Process management methods	Following a standard or commonly accepted methodology may influence the success of BPM initiatives	
Program and project management	BPM initiatives should be managed using effective program and project management practices. The efforts should be planned and controlled	Identifying and addressing the risks; involving the right people in the project; developing a process management plan; ensuring adequate financial resources; clearly defined objectives
Process measurement	Evaluating actual process performance through effective KPIs is important in translating strategic objectives to process-specific goals	set of key performance indicators (KPIs); benchmarking
Process related standards	The use of standards in structuring and defining processes and their outputs enable high-quality processes. Standards can be taken as reference sources in BPM implementations	Using the best modeling standards and techniques; implementation of internal and external standards

Source: Authors' own elaboration based on Tarhan and Turetken (2016); Buh et al. (2015);

Bai and Sarkis (2013); Hajiheydari and Dabaghkashani (2011)

The presented CSFs categories do not include any success factors characteristic of dynamic BPM, which are crucial not only in the case of knowledge-intensive organizations, but in the case of all organizations adapting to the current turbulent business environment and Industry 4.0, such as:

- The management of process roles in the scope of the actual empowerment of process performers to introduce changes to the standard process in the course of performance itself (performing limited experiments),
- The adaptation of the performed process to the requirements of a specific performance context by a performer with the necessary privileges,
- The on-line collection of information in event logs on levels IV and V according to the Process Mining Manifesto on all of the actions of the process performer and the full context of performance, including the actions undertaken through social media applications, e-mail, etc.,
- Machine learning on the basis of the analysis of the course of the performed process, including identified deviations from the standard process and their results,
- The distribution among the organization of knowledge verified, revealed, or created in the course of process performance.

In order for CSFs to truly reflect the main areas impacting or even determining the success of BPM implementation, they must be extended to include factors taking into account the nature of processes (predictability and repeatability of processes; complexity and contextuality of processes), and the intensity of the impact of knowledge management on the implementation and result of the process (the role of knowledge in the business process; process learning time/process knowledge acquisition time, knowledge scope and replacement rate).

### Analysis of currently used BPM MMs

The term maturity can be defined as “the ability to respond to the environment in an appropriate manner through management practices” (Bititci et al. 2015). Rosemann and de Bruin (2005a, b) describe maturity more pragmatically as “a measure to evaluate the capabilities of an organization in regard to a certain discipline.” Thus, organizational maturity reflects the adequacy of the organization’s practices in selected areas from the perspective of strategic goals and the business environment in which the organization operates. McCormack and Johnson (2001) and Skrinjar et al. (2008) empirically demonstrated a positive relationship between business process maturity and business performance (Van Looy et al. 2017).

Maturity models are multi-level frameworks describing a typical path of developing organizational capabilities (Poeppelbuss et al. 2011). Successive levels of maturity are defined in terms of evolutionary stages (Rosemann and de Bruin 2005a). In general, the development of a BPM MM has three primary aims (Van Looy 2010; Rosemann and de Bruin, 2005b):

1. To enable organizations to assess their current strengths and weaknesses in BPM (i.e., their as-is BPM position –mainly descriptive models),
2. To enable organizations to determine their desired maturity stage with respect to key factors within BPM (i.e., their to-be BPM position), and
3. To assist organizations in developing a BPM progress road-map to move from their as-is to their desired to-be positions (mainly prescriptive models).

Maturity models can be applied to both business processes and BPM implementation in organizations (vom Brocke et al. 2020; Dabaghkashani et al. 2012; Van Looy et al. 2011; Van Looy 2010; Rohloff 2009; Gartner 2008; Hammer 2007; Rosemann and de Bruin 2005a). BPM Maturity Models usually cover multiple dimensions such as governance, methods and tools, IT, and culture (Rohloff 2009; Rosemann and de Bruin 2005a; Rosemann et al. 2004). The lowest stage in the maturity model usually refers to the initial state of the organization in the analyzed area and is characterized by a lack of or basic capabilities in the analyzed field (e.g., business process management) (Becker et al. 2009). In contrast, the highest stage reflects “the state of being complete, perfect or ready” (Tarhan et al. 2015).

Organizations are constantly striving to raise their level of maturity, assuming that a higher level of maturity will translate into better results and value building (Van Looy et al. 2017). Therefore, maturity models can be used as a tool to improve the way organizations are managed, which allows them to deliver higher quality products and services (Tarhan et al. 2015; Rosemann et al. 2004).

Maturity models are extremely useful tools that can be used to assess the current situation, identify constraints, define improvement initiatives and control progress, guiding organizations in the process of evolution and adaptation to an increasingly demanding environment (Roeglinger et al. 2012; Becker et al. 2009; Kohlegger et al. 2009). Maturity models can also enable organizations to analyze compliance with industry standards, supporting them in defining priorities and achieving business goals (Lee et al. 2007). The proven, well-established requirements for BPM MMs in the literature on the subject are presented in Table 3.

**Table 3:** Requirements for BPM MMs

No.	The requirement for BPM MM	Source
1	Holistic view of the organization	Rosemann et al. (2004), Rosemann and de Bruin (2005b)
2	The possibility of assessing maturity, taking into account the criteria dedicated to non-traditional BPs	Chaghooshi, et al. (2016), Figueiredo et al. (2014), Rohloff (2009)
3	Configurability, taking into account the context of the organization's operation	de Bruin et al. (2005), Rosemann and de Bruin (2005a), Poeppelbuss and Roeglinger (2011), Van Looy (2013)
4	Multidimensionality of assessment	DeToro and McCabe (1997), Rosemann and vom Brocke (2015), Rosemann et al. (2004), Rosemann and de Bruin (2005a)
5	Multilevelness of assessed processes or analyzed criteria	de Bruin et al. (2005), Rosemann and de Bruin (2005b)
6	Possibility of cyclical repetition of the complete or partial assessment	Rosemann et al. (2004), Van Looy (2013)

Source: Authors’ own elaboration

The basis for most of the current BPM MMs is the Capability Maturity Model (CMM), proposed 30 years ago by the Software Engineering Institute at Carnegie Mellon University. It introduced the concept of five maturity levels (1. Initial, 2. Repeatable, 3. Defined, 4. Managed, 5. Optimizing) defined by special cumulative requirements (Roseman and vom Brocke 2015; Paulk et al., 1993). Smith and Fingar (2004) noticed as early as in 2004 that BPM MMs based on the CMM refer only to traditional, repeatable business processes and do not allow for the correct assessment of the process maturity of innovative organizations. In Industry 4.0, BPM maturity models must include dynamic, usually knowledge-intensive processes in order to support digital transformation and build competitive advantage. There were numerous attempts to solve this dilemma, based on the scheme proposed by CMM. According to the research by Van Looy et al. (2017), in 2017, there were already over 150 BPM MMs. Most BPM MMs adhering to the CMM formula have 5 or 6 levels of maturity (Rosemann et al. 2004; Paulk et al. 1993), at which the criteria or CSF are assessed in 5 or 6 areas (Rosemann and vom Brocke 2015). To some extent, this need has already been met in the new version of the CMMI model released by the CMMI Institute in 2018 by adding agile practices to the CMMI model (CMMI Institute 2017). Moreover, one of the goals of introducing the ISO 33000 family of standards in 2015 was to enable a more flexible adjustment of the process quality assessment framework to the current business requirements (ISO 2015a; ISO 2015b; ISO 2017). However, these changes did not bring about the expected practical results. The publications on this topic are dominated by articles presenting new models, theoretical articles, and articles comparing BPM MM. Less than 20% of publications referred to the practical application of BPM MMs, which means that BPM MM research has now become a theoretical field detached from practice (Tarhan et al. 2016). None of the available models propose the selection of evaluation criteria in accordance with the identified nature of the analyzed processes, so as to adapt the evaluation method to the actual nature of the processes and the BPM context (Szelągowski 2021; vom Brocke et al. 2014). This would allow for the full compliance of the maturity assessment criteria with the real nature of the business processes and CSFs that are relevant to the achievement of the organization's goals. For example, it would prevent the application in evaluation of the”repeatability” criterion to processes that require innovation and creativity, such as unpredictable research and development or sales processes.

**Fig. 1:**
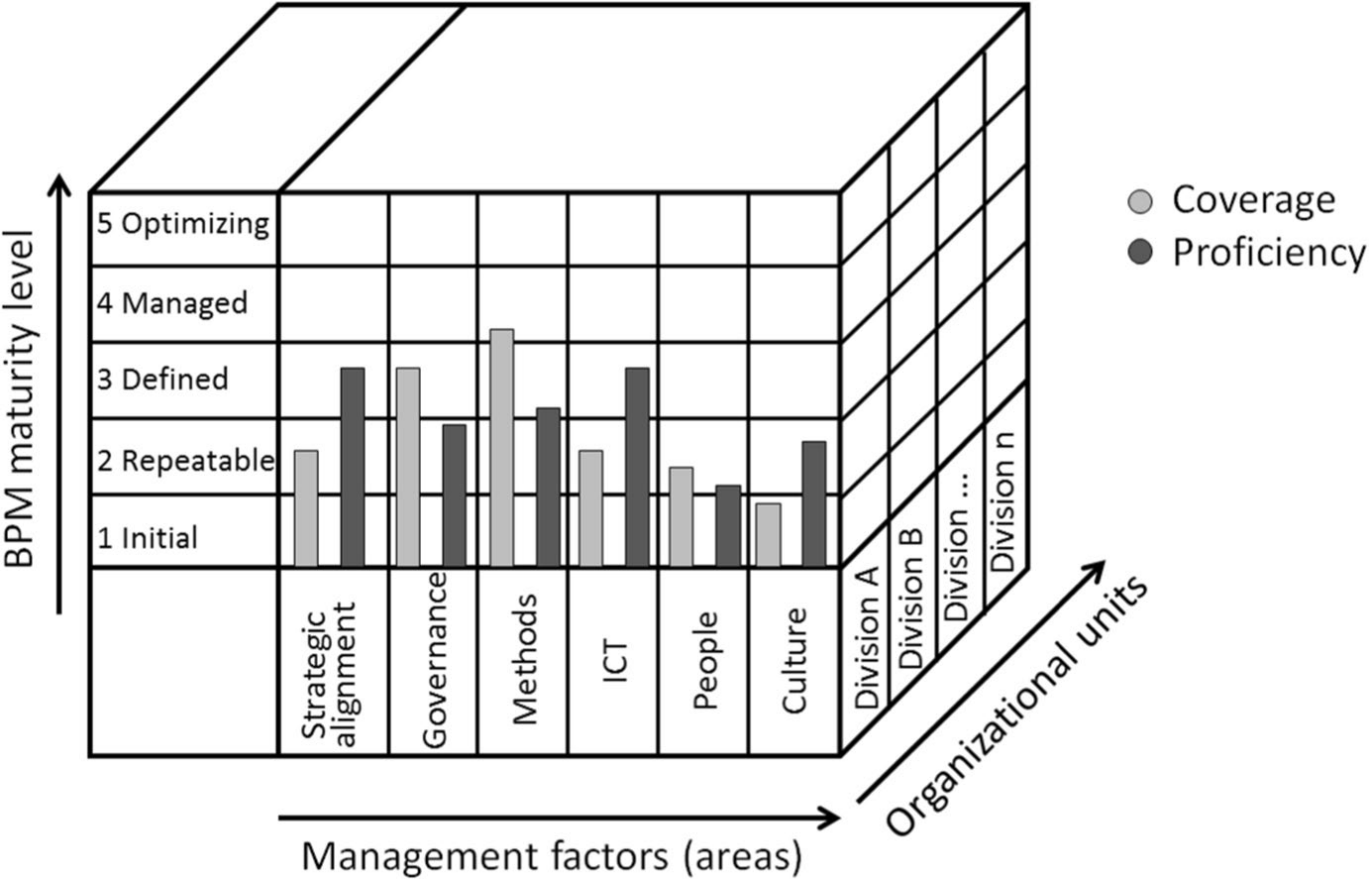
BPM Maturity Model according to Rosemann and de Bruin. Source: Authors’ elaboration based on Rosemann and de Bruin, (2005b), 16–19

An attempt to bring BPM MMs closer to practice and take into account the context of the operation of contemporary organizations was the model by Tonia de Bruin and Michael Rosemann, proposed in 2004–2006 (de Bruin 2009; Rosemann et al. 2006, 2004; Rosemann and de Bruin, 2005a, 2005b) (Fig. 1). In the scope of the proposed model, BPM maturity is defined as a combination of coverage and proficiency, which is similar to the notion of effectiveness and efficiency in the model by DeToro and McCabe (1997). Coverage refers to the level of implementation of BPM principles, whereas proficiency measures the quality and effectiveness of BPM within the organisation. In other words, coverage asks how far throughout the organisation BPM activities extend and proficiency asks how well BPM activities are conducted. Attaining a higher maturity stage requires improvement in both “coverage” and “proficiency.”

It assumed the study of five dimensions of the organization's process maturity, while the Factor dimension itself was de facto a multidimension, which consisted of six separate areas, each of which should be assessed according to multiple criteria. In order to ensure the flexibility and detail of the model expected by the users, it was possible to define sub-factors by the researchers that did not complicate the basic shape of the model, but allowed for the assessment to be detailed in accordance with the current context of the organization's operation. This enabled the assessment to be refined in areas considered by the investigators to be important to the ongoing operation or development of the organization. For example, sub-factors for the factor People may be: knowledge, capabilities, education, training, and skills (Rosemann and de Bruin 2005a).

The model enables the definition of sub-factors, which allows for its free customization to the current needs of the organization. However, it also enables the assessment of BPM maturity within individual organizational units, not within processes, which, after all, can run through many units. In other words, it is still not a coherently integrated element of the BPM implementation (i.e., the model assumes the analysis of processes within individual organizational units, not within processes that run across unit boundaries), nor is it integrated with the BPM Lifecycle.

The problem of balancing the detail and complexity of BPM MM and its adaptation to the context of the organization's operation was solved by the creators of BPMM-OMG. They proposed three groups of criteria, including 351 examined criteria for all maturity levels. At the same time, they allowed the researchers to exclude criteria from the study in areas that will not be included in the study. The reason for the exclusion may be, for example, the lack of a specific group of processes in the organization or the recognition of their study as not bringing significant value to the study (OMG 2008). As in the Rosemann and de Bruin (2005a) model, it is extremely important to select research areas in accordance with the context of operation and the needs of the organization under study. However, they still do not solve the problems related to the use of BPM MMs and their excessive complexity, making their use extremely difficult (Szelągowski and Berniak-Woźny 2019; Maull et al. 2003).

## Results

### The proposal for a BPM maturity assessment framework

According to a literature review conducted by Van Looy et al. (2017), organizations could choose a process maturity model from among over 150 known BPM MM. As shown above, they proposed various approaches to the selection of evaluation criteria: from the unrestrained decisions of people conducting the research (e.g., Rosemann and de Bruin 2005b), to choosing from the usually long list of criteria proposed by the authors of the model (e.g., OMG 2008). They even allowed for building rules and criteria from the bottom up in individual business units or in the case of isolated projects (Rosemann et al. 2004). However, none of them proposed to objectify the selection of criteria according to the actual context of the organization's operation (Van Looy 2013); e.g., by linking the scope of the assessment to processes that create value for the customer or generally identified processes in the organization (vom Brocke et al. 2014).

**Fig. 2:**
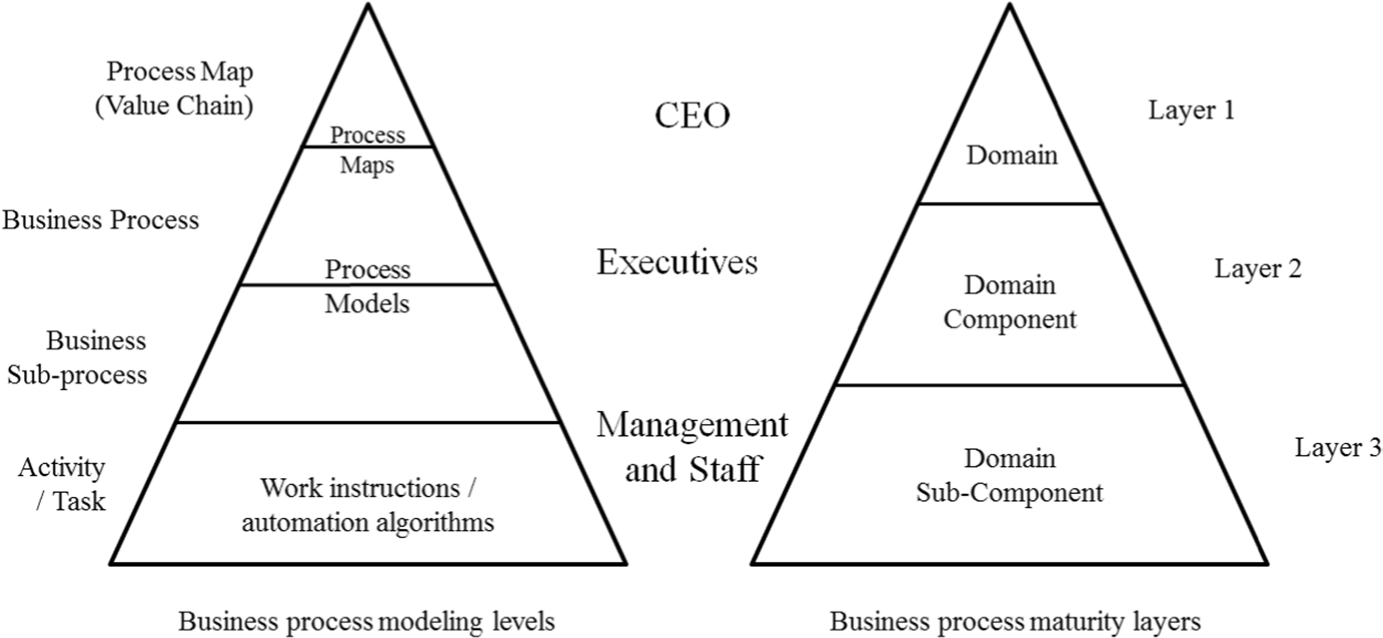
Business process modeling levels and business process maturity layers. Source: Authors’ elaboration based on Mahal (2010) and de Bruin et al. (2005)

The preparation of process maps at the highest level ("N") and one step below in detail ("N-1") is one of the first generally recommended steps for BPM implementation (Hammer 2015; Mahal 2010; Burlton 2001). Usually, N and N-1 level maps are prepared first and then they are detailed to the N-2, N-3, and even N-4 level process models depending on the goals and scope of implementation, organizational needs, etc. Assessment of the organization's process maturity, synchronized with modelling as part of an in-depth analysis of the organization's processes, enables recognition of the nature of the processes and requirements for their implementation, in accordance with the needs of the implementation (Fig. 2) (Mahal 2010; de Bruin et al. 2005).

The data prepared during process maturity assessment can immediately be taken into account when planning and performing BPM implementation. An additional advantage is that the synchronization of these works reduces their total labour-intensity thanks to the fact that the teams responsible for the execution of these works gather and “enter” the subject of individual mega-processes or processes once, and not twice at separate meetings.

The authors propose to extend BPM maturity assessment in an organization so that the assessment takes into account the diverse natures of BP and BPM context in contemporary organizations operating in Industry 4.0 and digital transformation as objectively as possible, and at the same time fits into the empirically verified methods of BPM implementation. Of course, it is not about rejecting the results of previous works dedicated to traditional BPM, but about extending a concept in which traditional BPM and the corresponding BPM MMs will be treated as a special case (Heller 2014, 50–51).

This concept includes using standard BPM Lifecycle phases (such as “Defining goals,””Preparation of the project” (Szelągowski 2018), or “Process identification” and “Process discovery” (Dumas et al. 2018)) to obtain objective data on:

- BPM CSF in the organization,
- The nature of the processes actually carried out by the organization.

**Fig. 3:**
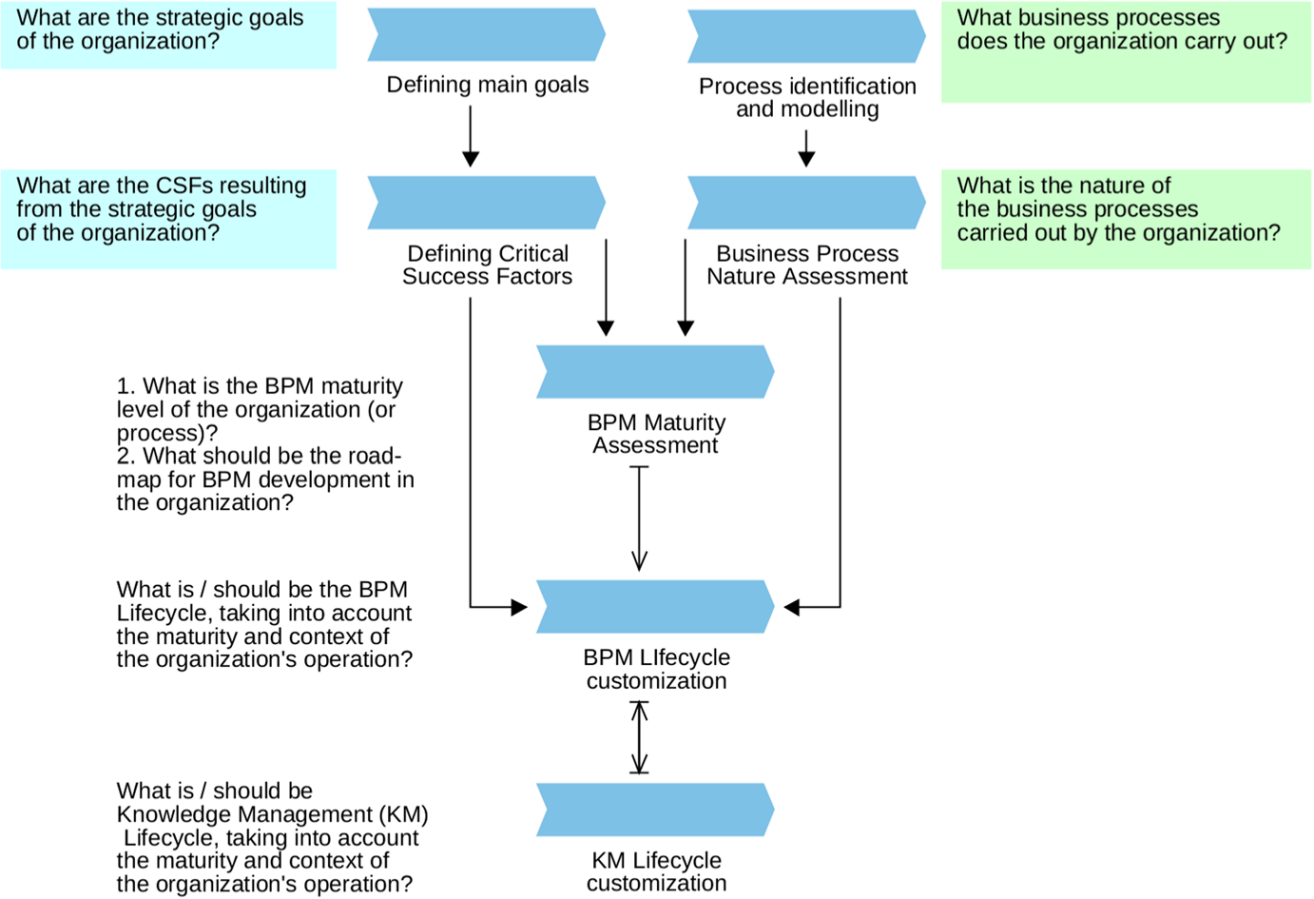
The BPM Maturity assessment process map – a block diagram. Source: Authors’ own elaboration

The framework embedding BPM maturity assessment in the implementation of BPM in the organization is presented in Fig. 3. As in any implementation or update of BPM, the framework assumes in the first phase defining the goals of BPM implementation and their operationalization by CSF in accordance with the current strategy of the organization. Then, in practice usually in parallel, the identification of processes at the N and N-1 levels is carried out (most often as part of the preparation of organization process maps and general models) along with the assessment of the nature of BP in the real context of the functioning of the organization. Only when the above data is available is the process maturity assessment performed, taking into account both the nature of business processes and CSF resulting from the organization's strategy. The results of the BPM maturity assessment are the basis for the further implementation of BPM, constituting the basis for the development based on objective data:

- Comparison (benchmarking) with market data (e.g. industry data),
- Recommendations regarding, for example, IT systems or human resource management supporting the implementation of processes,
- Verification of the selected implementation methodology and preparation of a roadmap for the development and improvement of the organization's BPM.

They also constitute the basis for the analysis of the requirements for the life cycles of individual process groups depending on their nature and the BPM maturity of the organization: from the traditional BPM life cycle based on the Deming cycle to the BPM life cycle for kiBPs, taking into account the need for the possible integration of BPM Lifecycle with the knowledge life cycle management.

The inclusion of process maturity assessment in the BPM implementation and next process management makes it natural to use advanced IT tools supporting the operation in individual phases. For example, in the Process Identification and modeling phase, process mining tools and process modelers should be used, and in the Defining main goals and Defining CSF phases, tools supporting the management of the organization's strategy and strategic goals (e.g. based on the Balance Scorecard) integrated with business intelligence. As part of the BPM Maturity Assessment phase, solutions in the field of process mining, workflow management, and artificial intelligence should be used, and above all, solutions for communication and information gathering.

The BPM MM assessment process presented above assumes the possibility of assessing all or selected processes of the organization. Therefore, it can be used not only for assessment as part of the upcoming BPM implementation, but also for repeated assessments of selected mega-processes or processes. Reassessments of BPM maturity are inevitable due to the speed of changes in the business environment, available and used technologies, or social culture and work culture, which may cause changes in the course, importance, and even the nature of the business processes or strategic goals of the organization. Therefore, before each repeated assessment, the strategic goals, and CSFs of the assessed BPs should also be verified and updated and each assessment should be followed by a verification of whether there is a need to change the BPM Lifecycle of the assessed processes.

According to the principles of creating the organization's process architecture, the goals of the Core processes and the goals of implementing BPM in the organization must be consistent with the strategic goals of the organization. Then, the CSFs created on that basis and used to assess the process maturity of the organization will also be directly consistent with the strategic goals. The process maturity assessment will therefore be strictly harmonized with the current management. For traditional business processes, CSFs will conform to the requirements for providing a traditional, Deming Cycle-compliant BPM Lifecycle, in which knowledge is used to improve processes beyond the time of execution. However, for semi-structured and unstructured processes, especially for kiBP, it is important to define maturity, taking into account that these processes create value through the use, verification, and creation of knowledge, not outside of, but during the implementation of the processes. The more unpredictable the processes are and the more knowledge-intensive, the more the criteria or CSF should be taken into account when assessing maturity, other than those applied to traditional BP. BPM MM dedicated to Knowledge Intensive Industry or Knowledge Intensive Organization or kiBPs must take into account the nature of the processes, including the degree of their unpredictability and knowledge-intensity. Since different groups of processes within a single organization may have different natures (even in innovative startups processes must be implemented in accordance with applicable law, which means there exist fully predictable accounting or employee employment processes), the assessment criteria should be detailed to level N- 1 and sometimes N-2. This may require the use of different criteria for different groups of processes (Makani and Marche 2010). Therefore, when assessing the BPM maturity of an organization, criteria or CSF relating to unpredictable and knowledge-intensive BPs must appear at individual levels and may be different for processes or groups of processes of a different nature. Moreover, as the unpredictability and intensity of BPs knowledge increases, the criteria and CSF relating to the assessment of semi-structured and unstructured processes should have an increasing impact on the overall assessment of BPM maturity in an organization.

**Fig. 4:**
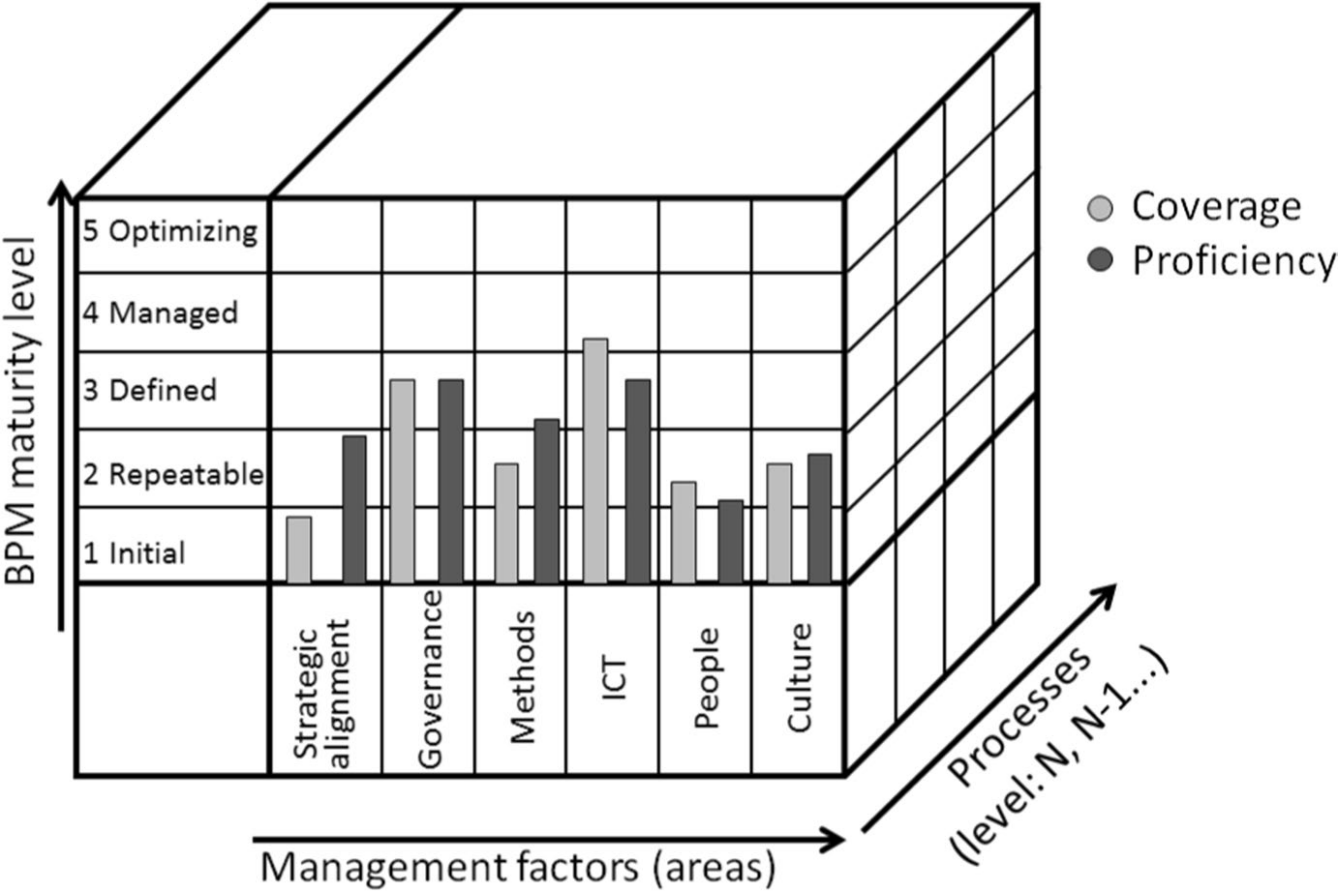
BPM Maturity assessment space taking into account criteria dedicated to BPs of different natures. Source: Authors’ own elaboration

The BPM Maturity assessment process presented in Fig. 4 allows, in accordance with the framework of general design principles for maturity models by Roeglinger et al. (2012, 5–7), for the preparation of intersubjective assessment criteria for each maturity level, and at the same time ensures their compliance with the actual context of implementation, among others processes and the strategic goals of the organization. If the nature of different groups of processes implemented by the organization is different, then the correct assessment of their maturity will require the use of various criteria and may lead to the recommendation of various BPM lifecycles, and even the incorporation of different knowledge management solutions for different groups of processes in a single organization (different KM Lifecycle) (Makani and Marche 2012).

### BPM Maturity assessment space proposal

Taking into account the previously discussed requirements for integrating the maturity model with BPM implementation methodologies, as well as the dependence of the maturity criteria on the nature of the processes implemented in a specific context of the organization's operation, it is necessary to:

- Separate conducting BPM maturity assessment for groups of processes, e.g., according to organization process maps identified at the N or N−1 level,
- Differentiate BPM maturity assessment criteria depending on the nature of business processes.

As shown in Table 4, this requires the differentiation of assessment criteria according to the nature of business processes (among others unpredictability and knowledge-intensity). Assuming a standard 4-level scale of this dimension, it increases e.g., in the Rosemann and de Bruin (2005b, 16–19) BPM MM the total number of criteria from 12 to 48. However, this does not mean increasing the workload of the study itself, because only the criteria dedicated to processes with a certain kind will be used when examining each group of processes. The assessment will therefore continue to be carried out using the same number of criteria (in our example – 12), but these criteria will be much better suited to the nature of the organization's activities and the challenges it faces. Of course, it is possible for individual organizations or industries to create complementary, additional sub-factors and corresponding criteria. These sub-factors can be different for BPs of different natures and can change over time depending on the needs and challenges of the organization.

**Table 4:**
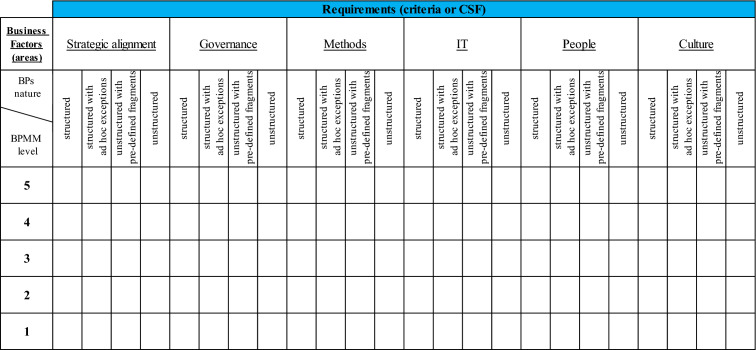
The general BPM maturity assessment criteria definition form for BPs of different natures

Source: Authors’ own elaboration

The result of the BPM maturity assessment in the organization or each group of processes or even each of the processes will be 6 evaluations in Rosemann and de Bruin BPM MM 12 evaluations, two for each of the 6 factors). They can be described as 12 points in the rating space, described by the coordinates (Process, Factor, Maturity) (Fig. 4). This allows for any analysis according to the dimensions corresponding to individual factors or according to process groups. In addition, the scope of possible analyses and their detail will increase if the assessment is extended with additional sub-factors or the dataset contains additional information (e.g., weight or nature of the process).

Both to facilitate the process maturity assessment and the analysis of its results, an application enabling the assessment of BPM Maturity would be extremely helpful (Rosemann and vom Brocke 2015; Tarhan et al. 2015; Rosemann and de Bruin 2005b). It should, as in the case of Tarhan et al. (2015) or the Rosemann and de Bruin (2005a) model, enable the selection of the scope and parameters of the study in accordance with the needs of the organization. According to the framework, the first step is to define the context and criteria for the study (Fig. 4). Only in the second stage should the actual maturity study be adapted to the context of the organization's operation, in the first approximation with regard to the nature of the processes, in the next ones – the industry or the size of the organization.

The proposed presentation method of the results of the analysis uses a clear, rich in information, and interactive visualization format for the presentation of the process maturity assessment proposed by Roseman and de Bruin (2005a). However, it is a visualization of the results of the study conducted in accordance with Table 4 (and thus also includes processes that require dynamic management – semi-structured and unstructured) and with the BPM Maturity assessment process map from Fig. 3, i..e. not in isolation, but as part of the BPM implementation or the ongoing BPM.

Despite the similar form of presentation, the aim is to present an assessment of BPM maturity from the point of view of modern business, without the defect of applying assessment criteria appropriate for traditional, repeatable business processes to processes that require dynamic management. Thus, the innovation in the form of presenting the results is the going-away from the evaluation of processes within organizational units (silos) to the evaluation of business processes (end-to-end), which is imposed in the presentation of the assessment of business processes. This enables, in line with the needs of the organization, an increasingly detailed analysis of BPM maturity, not limited to analysis within individual organizational units. This is particularly important considering that the greatest problems with the smooth implementation of business processes occur when they cross the boundaries of organizational units.

## Conclusion

BPM MMs allow for the assessment of the current BPM maturity of the organization and/or individual groups of processes and, on this basis, comparison with the competition and planning of further management activities. They are extremely important to the success of organizations, especially at the time of rapid digitization or automation and/or organizations changing their model to e-business (Bispo et al. 2019). However, it is becoming increasingly clear that the development of the BPM MM has not kept up with changes in the entire business ecosystem. The sheer number of over 150 BPM MMs points to an extremely hectic search for practically useful BPM MMs (Van Looy et al. 2017). However, the significant dominance of conceptual and review publications over publications on the practical applications of BPM MM shows that this search has been a challenge for years. The research presented in the article indicates three reasons for this:

1. Failure to take into account the nature of BP in the context of the organization's operation, causing most BPM MMs to take into account only traditional BPs and their BPM Lifecycle in a situation where in modern organizations these processes account for only about 30% and less important BPs,
2. Lack of approach to BPM maturity research from the user's point of view and embedding it in generally accepted BPM implementation methodologies (Tarhan et al. 2015). As a consequence, no assessment method was developed that would meet the practical requirements of contemporary business, such as depth and detail of the evaluation, low evaluation effort (low human-intensive process), the possibility of systematic repetition of evaluations, comparing their results and preparing business-understandable recommendations based thereon.
3. Lack of intuitive, ergonomic software to conduct process maturity assessments as part of a wider implementation process (Tarhan et al. 2015). It has been postulated for over 15 years that there is a need for balance between complexity (the truthfulness of the image generated by the model) and readability (simplification to ensure the comprehensibility of the image generated by the model) (Roseman and de Bruin 2005b).

The framework presented in the article is a continuation of, and at the same time clearly differs from, the tradition of building BPM MMs based on the CMM model. The model proposes the use of a clear and understandable system of 5 levels of maturity measured in 6 main areas of BPM. At the same time, it takes into account different requirements for processes of different natures and proposes a strict connection of the BP assessment criteria with the CSFs that agree with the strategic goals of the organization. The framework proposes a two-stage, comprehensive process of organizational process maturity assessment, fully integrated with the BPM implementation process and further business process management. The presented concept allows organizations in which dynamic kiBPs play an important role in creating value to assess process maturity.

The presented concept enables organizations in which dynamic kiBPs play an important role in value creation to perform a much more realistic and reliable assessment of process maturity than the current BPM MM models. From a practical point of view, it is extremely important that the data provided by the proposed assessment framework are much more nuanced and, in which is not available in the CMMI model, show the maturity of processes at the N or N-1 levels, and even lower if needed. Thanks to this, the management team obtains a more realistic, but also, if necessary, a more detailed picture of the process maturity of the organization, which allows organizations to make decisions in line with their actual maturity and needs.

It is also important to include the process maturity assessment of the organization in the standard methodology of BPM implementation. As a result, process maturity assessment is not an additional effort and cost artificially attached to implementation, and is not perceived by the participants as an additional, tedious, or even unnecessary obligation. In the presented framework, maturity assessment is a standard element that has a significant impact on the preparation and implementation of BPM. For organizations process-managed in accordance with the framework, the maturity assessment becomes a standard management element, enabling a better adjustment of BPM goals and methods to their goals, potential, and current needs.

The results presented in this article are the starting point for future research and development projects. The practical goal of future research will be to develop a detailed methodology and create a tool for BPM maturity assessment and BPM MM presentation. Ultimately, its integral part will be a knowledge base containing recommendations and roadmap proposals or comparative indicators for individual groups of processes, varied for industries, the size of the organization, and the actual nature of business processes in a specific context of operation and the level of maturity of the organization. This will require looking at BPM MMs as a constantly updated tool for conducting BPM development in the organization, and not just for a one-time examination as part of the implementation preparation. Therefore, in the plans for future research, the authors take into account, inter alia, practical preparation (or supplement) of the following:

- Methodology for determining the criteria for assessing process maturity depending on the context of the organization's operation, including criteria taking into account the dynamism of action and the use of knowledge,
- Methodology of selecting and implementing or updating IT systems supporting the implementation of BPM depending on the BPM maturity and context of the organization's operation,
- Case studies illustrating the practical application and usability of the above-mentioned methodologies, methods, and tools,
- Continuous verification and updating of recommendations, including recommendations in the areas of HRM and ICT.
